# Retrospective Study on Short-Term Reverse Cardiac Remodeling in Obese Patients Undergoing Sleeve Gastrectomy

**DOI:** 10.3390/jcdd11120389

**Published:** 2024-12-03

**Authors:** Carmine Izzo, Valeria Visco, Alessandra Cirillo, Davide Bonadies, Giuseppe Caliendo, Maria Rosaria Rusciano, Nicola Virtuoso, Francesco Loria, Alessia Bramanti, Eleonora Venturini, Paola Di Pietro, Vincenzo Pilone, Luigi Schiavo, Albino Carrizzo, Carmine Vecchione, Michele Ciccarelli

**Affiliations:** 1 Department of Medicine, Surgery and Dentistry, University of Salerno, 84081 Salerno, Italy; vvisco@unisa.it (V.V.); alessandra.cirillo167@gmail.com (A.C.); d.bonadies1@gmail.com (D.B.); giuseppecaliendo1995@gmail.com (G.C.); mrusciano@unisa.it (M.R.R.); francescoloria94@gmail.com (F.L.); abramanti@unisa.it (A.B.); pdipietro@unisa.it (P.D.P.); lschiavo@unisa.it (L.S.); acarrizzo@unisa.it (A.C.); cvecchione@unisa.it (C.V.); mciccarelli@unisa.it (M.C.); 2 Cardiology Unit, University Hospital “San Giovanni di Dio e Ruggi d’Aragona”, 84081 Salerno, Italy; n1virtuoso@hotmail.it; 3 Vascular Physiopathology Unit, IRCCS Neuromed Mediterranean Neurological Institute, 86077 Pozzilli, Italy; ele.venturini94@gmail.com; 4 Public Health Department, Naples “Federico II” University, AOU “Federico II”, Via S. Pansini 5, 80131 Naples, Italy; vincenzo.pilone@unina.it

**Keywords:** obesity, echocardiography, cardiac remodeling, cardiology, cardiovascular risk factors, bariatric surgery, sleeve gastrectomy, weight loss

## Abstract

Severe obesity is closely associated with an increased risk of comorbidities and alterations in cardiac structure and function. The primary objective of this study was to investigate cardiovascular (CV) risk factors and ventricular remodeling in individuals from an obese population eligible for bariatric surgery. The secondary objective was to evaluate changes in anthropometric, clinical laboratory, and echocardiographic measurements 12 weeks after surgery compared to baseline values. This retrospective observational cohort study involved patients from a single specialized bariatric surgery center. A total of 35 patients were included (mean age 41.5 ± 10.3 years; BMI 43.4 ± 6.6 kg/m^2^), of whom 34.2% had a family history of coronary artery disease (CAD), 5.7% had a prior history of CAD, 8 had essential hypertension, 11.4% had dyslipidemia, 20% were smokers, and 8.6% were former smokers. Approximately 57% of the patients exhibited concentric left ventricular remodeling, and 14% had grade I diastolic dysfunction. At 12 weeks post-surgery, with an average weight loss of 25 kg and a mean BMI reduction of 8.5 kg/m^2^, 14% of the patients still exhibited concentric left ventricular remodeling, and about 11% had grade I diastolic dysfunction. Bariatric surgery contributes to the improvement of cardiac function and structure over time as a result of significant weight loss.

## 1. Introduction

### 1.1. Obesity

Obesity, defined by the World Health Organization (WHO) as excessive body fat accumulation, arises from genetic, environmental, and lifestyle factors, including the adoption of a sedentary “Western lifestyle” and high-calorie diets. This global public health challenge has arisen in recent decades due to socioeconomic and behavioral shifts [1,2].

The Body Mass Index (BMI) is the standard tool for classifying obesity, with thresholds of 18.5–24.9 kg/m^2^ for normal weight, over 25 kg/m^2^ for overweight, and over 30 kg/m^2^ for obesity. Severe obesity is marked by a BMI exceeding 40 kg/m^2^. However, BMI has limitations, as it fails to account for differences in body composition, such as muscle mass and fat distribution, potentially misclassifying individuals [3,4].

Advanced methods like Dual-Energy X-ray Absorptiometry (DEXA), Bioelectrical Impedance Analysis (BIA), and imaging techniques provide more precise assessments but are rarely used clinically due to complexity. Instead, simpler measurements like waist circumference and metabolic indicators (e.g., blood glucose and cholesterol levels) are preferred to evaluate health risks more comprehensively [5].

This nuanced approach acknowledges the rising global prevalence of obesity driven by decreased physical activity and dietary changes, personalized lifestyle modifications to address associated health risks effectively [4,6].

### 1.2. Obesity and Left Ventricular Remodeling

The heart exhibits remarkable adaptability, remodeling itself in response to physiological and pathological conditions. Physiological remodeling occurs during physical exercise, pregnancy, or growth, whereas pathological remodeling, such as left ventricular hypertrophy (LVH), arises from chronic conditions like hypertension. LVH involves molecular and cellular changes, including myocyte enlargement and extracellular matrix restructuring. Initially compensatory, these adaptations may become maladaptive, contributing to cardiovascular complications [7,8].

Hypertension is a primary driver of cardiac remodeling, triggering mechanical stress on ventricular walls, collagen turnover, and calcium transport dysfunction in myocytes. Elevated intracellular calcium activates calcineurin, leading to hypertrophic signaling. Echocardiography remains the gold standard for assessing left ventricular structure, with left ventricular mass index (LVMI) and relative wall thickness (RWT) used to classify remodeling patterns into concentric or eccentric hypertrophy and concentric remodeling [9,10].

Obesity is strongly linked to left ventricular remodeling and heart failure risk. Excess weight imposes hemodynamic and metabolic burdens, leading to structural and functional changes in the heart. Bariatric surgery, particularly sleeve gastrectomy (SG), has emerged as an effective treatment for severe obesity, offering not only significant weight loss but also cardiovascular benefits. Studies indicate that weight loss achieved through bariatric surgery can reverse cardiac remodeling, reducing left ventricular mass, wall thickness, and improving systolic and diastolic functions.

Research by Frea et al. and Karason et al. demonstrates that bariatric surgery alleviates concentric hypertrophy and improves diastolic filling by lowering hemodynamic load and enhancing metabolic health. Systematic reviews and meta-analyses corroborate these findings, highlighting reductions in LV wall thickness and improvements in ventricular compliance and function after surgery. The mechanisms of reverse remodeling are multifactorial, involving hemodynamic, inflammatory, and metabolic changes [10,11,12].

Bariatric surgery reduces preload and afterload on the left ventricle, alleviating myocardial wall stress. It also decreases systemic inflammation and oxidative stress, as evidenced by reductions in inflammatory markers like C-reactive protein (CRP) and interleukin-6 (IL-6), both associated with LV hypertrophy and fibrosis. Enhanced glucose metabolism and insulin sensitivity following surgery further support cardiac function by reducing myocardial fat infiltration, which is linked to impaired relaxation and compliance [13].

Moreover, weight loss lowers circulating levels of leptin and other adipokines that activate the sympathetic nervous system, potentially reducing heart rate, blood pressure, and myocardial workload. These suggest improvements that bariatric surgery not only promotes weight reduction but also improves cardiac health, particularly for individuals with obesity-related left ventricular remodeling [14].

The clinical significance of addressing LVH lies in its association with increased risks of heart failure, arrhythmias, myocardial infarction, and sudden cardiac death. By mitigating these risks, bariatric surgery emerges as a transformative approach for both obesity and cardiovascular health, offering hope for improved outcomes in patients with obesity-related cardiac remodeling [15,16].

### 1.3. Obesity Therapy and Treatment Options

Managing obesity is a persistent challenge, with lifestyle modifications like a healthy diet and regular exercise serving as the cornerstone of treatment. A weight loss of at least 5% from baseline is associated with significant improvements in cardiometabolic risk factors. However, long-term adherence to lifestyle changes is difficult, with most individuals regaining weight within five years. While medical therapies offer limited success, bariatric surgery remains the most effective long-term treatment for sustainable weight loss and the alleviation of obesity-related complications [17].

Bariatric surgery is recommended for individuals with a BMI ≥ 40 kg/m^2^ or those with a BMI ≥ 35 kg/m^2^ accompanied by comorbidities such as type 2 diabetes, hypertension, dyslipidemia, non-alcoholic fatty liver disease, or severe sleep apnea. Candidates must demonstrate prior failure with conventional treatments and undergo comprehensive evaluations to ensure readiness and commitment to postoperative care [18,19].

Surgical techniques are categorized as malabsorptive, which limit nutrient absorption, or restrictive, which reduce stomach capacity to promote satiety. Common procedures include Roux-en-Y gastric bypass, sleeve gastrectomy, and adjustable gastric banding, which lead to substantial weight loss sustained over a decade [20]. Beyond weight reduction, bariatric surgery improves cardiovascular health by mitigating risk factors and reversing structural cardiac changes [21].

This study evaluates cardiovascular risk factors and echocardiographic alterations in obese individuals, exploring how these parameters are modified after bariatric surgery to advance understanding of its benefits for obesity-related cardiovascular health.

## 2. Materials and Methods

### 2.1. Study Design and Population

This is a retrospective observational cohort study involving obese patients who were indicated for bariatric surgery via sleeve gastrectomy (SG) at the Azienda Ospedaliera Universitaria San Giovanni di Dio e Ruggi d’Aragona in Salerno. The patients were recruited between June 2022 and April 2023 in accordance with the guidelines of the Italian Society of Surgery (SIC) and the Italian Society of Obesity Surgery (SICOB). The enrolled patients were referred from the Azienda Ospedaliera Universitaria San Giovanni di Dio e Ruggi d’Aragona in Salerno. All patients referred were included in the study regardless of age if they fell within previously mentioned guidelines. Exclusion criteria included previous major cardiovascular disease or other concomitant/previous major disease (e.g., oncological disease).

Data were collected during the preoperative assessment, which was conducted on a day-hospital basis at the Azienda Ospedaliera Universitaria San Giovanni di Dio e Ruggi d’Aragona. The collected data included anthropometric measurements, resting vital signs, instrumental parameters, electrocardiographic readings, and transthoracic echocardiographic assessments. Hematochemical values were retrieved through the hospital’s online information system.

### 2.2. Clinical Examination, Laboratory Testing, and Instrumental Testing

All patients had the following measurements recorded: age, sex, height, weight, waist and hip circumference, and BMI. Height was measured using a wall-mounted tape measure with patients standing barefoot and with feet together. Weight was measured using a KERN platform scale (model MPO 300k-1LM, Frankfurt am Main, Germany) with patients wearing light clothing and no shoes. Waist circumference was measured with a non-flexible tape measure placed midway between the lower rib margin and the iliac crest, encircling the entire waist. Hip circumference was measured with a standard non-flexible tape measure placed around the widest part of the hips, encircling the entire circumference. BMI was calculated as weight (kg) divided by the square of height (m^2^). Blood pressure was measured three times within a 10–20-min interval using the same aneroid sphygmomanometer (ERKA, model 1-tube EN 1060 Kobold Smart Rapid with a size 6 Adult Large cuff 34–43 cm, Bad Tölz, Germany). Measurements were taken with the cuff covering two-thirds of the left arm while the patient was seated. The average of the results was calculated. Systolic blood pressure was defined as the value at which the sound begins, and diastolic pressure as the fifth Korotkoff phase. Values were recorded in mmHg.

ECG was performed to assess cardiac electrical activity using standard 12-lead methods with the MAC2000 (GE Healthcare, Waukesha, WI, USA) and was interpreted by experienced operators. Echocardiographic assessments were conducted to evaluate ventricular structure and function using standard methods [16]. Two of the same expert operators, both medical director cardiologists, not blinded, performed the echocardiograms using the Vivid E9 scanner (GE Healthcare, Waukesha, WI, USA) with offline analysis to ensure consistency (EchoPac version 201, GE Healthcare, Waukesha, WI, USA), equipped with a 4.6 MHz transducer (GE-M5Sc-D XDClear, GE Medical Systems, Waukesha WI, USA). Measurements were performed according to the guidelines and recommendation.

Echocardiography enables accurate assessment of left ventricular remodeling in patients. The study of ventricular geometry begins with the measurement of interventricular septum thickness (IVS), posterior wall thickness (PW), and left ventricular end-diastolic diameter (LVEDd). These measurements allow for the calculation of ventricular volume and mass using specific formulas. Currently, left ventricular mass (LVM) is calculated according to the recommendations of the American Society of Echocardiography (ASE) [22].

Normal values for left ventricular mass indexed to body surface area (BSA) should be less than 95 g/m^2^ for women and 115 g/m^2^ for men, according to linear methods. Left ventricular hypertrophy (LVH) is defined when left ventricular mass index (LVMI) values exceed 115 g/m^2^ for men and 95 g/m^2^ for women. The calculation of relative wall thickness (RWT) allows for the classification of hypertrophy as concentric (RWT > 0.42) or eccentric (RWT ≤ 0.42) and helps identify concentric remodeling without hypertrophy (normal LVMI with increased RWT). This method identifies four geometric patterns: normal (LVMI ≤ 115 g/m^2^ for men and ≤95 g/m^2^ for women; RWT ≤ 0.42); concentric ventricular remodeling (LVMI ≤ 115 g/m^2^ for men and ≤95 g/m^2^ for women; RWT > 0.42); eccentric ventricular hypertrophy (LVMI > 115 g/m^2^ for men and >95 g/m^2^ for women; RWT ≤ 0.42); and concentric ventricular hypertrophy (LVMI > 115 g/m^2^ for men and >95 g/m^2^ for women; RWT > 0.42) [22].

Laboratory tests in fasting venous blood samples were analyzed for triglycerides (TGs), low-density lipoproteins (LDLs), high-density lipoproteins (HDLs), total cholesterol, glucose, glycated hemoglobin (HbA1c), complete blood count, hemoglobin, C-reactive protein (CRP), erythrocyte sedimentation rate (ESR), N-terminal pro b-type natriuretic peptide (NT-proBNP), albumin, creatinine, urea, uric acid, sodium, potassium, calcium, aspartate aminotransferase (AST), alanine aminotransferase (ALT), and total bilirubin. The analyses were performed at the laboratory of the Gaetano Fucito Hospital in Mercato San Severino (SA).

## 3. Statistical Analysis

The statistical analysis of the collected data was performed using both parametric and non-parametric tests as appropriate. Specifically, the Student’s *t*-test and Mann–Whitney test were used for continuous variables, while Fisher’s exact test was employed to compare frequencies and categorical variables. Before applying the appropriate tests, a Kolmogorov–Smirnov (K–S) “Goodness of Fit” test was conducted to assess whether the continuous variables followed a normal distribution. Simple linear regression was used to estimate the relationship between one independent quantitative variable and one dependent quantitative variable of interest. Measures of central tendency and dispersion were calculated, including means, standard deviations, and medians. Additionally, inferential statistics were employed to determine the probability of obtaining observed differences by chance alone. Statistical analyses were carried out using GraphPad^®^ version 9.5.5 (La Jolla, CA, USA) for Macintosh^®^. Statistical significance was defined as *p* < 0.05 in a two-tailed test with a 95% confidence interval.

## 4. Results

Thirty-five obese patients were evaluated at baseline (T0) and 12 weeks post-bariatric surgery (T1). At T0, the mean age was 41.5 years, with a mean weight of 124.1 kg, BMI of 43.5 kg/m^2^, waist circumference of 127.7 cm, and hip circumference of 136.5 cm. By T1, these values improved significantly, with mean weight at 99.6 kg, BMI at 35.03 kg/m^2^, waist circumference at 108.5 cm, and hip circumference at 118.2 cm. Patients showed an average weight loss of 24.46 kg, equivalent to a BMI reduction of 8.47 kg/m^2^ (Table 1).

At baseline, 34% of patients reported a family history of coronary artery disease (CAD), 22% had hypertension, and 11% had mixed dyslipidemia. Smoking was reported by 20% (current) and 8.5% (former). Additionally, 57% had undiagnosed dyslipidemia, 29% had elevated blood pressure, and 28.5% met metabolic syndrome criteria. By T1, only one patient had elevated blood pressure, and dyslipidemia persisted in four patients, reflecting significant improvements in cardiovascular risk factors (Table 2).

Laboratory findings demonstrated notable improvements in lipid profiles, glucose metabolism, and inflammatory markers. LDL levels reduced significantly, while HbA1c and uric acid levels improved, supporting metabolic benefits from weight loss. Echocardiographic measures, including lateral E’ wave and E/e’ ratio, indicated enhanced diastolic function. Importantly, no patients exhibited impaired glucose tolerance or metabolic syndrome at follow-up (Table 3).

**Figure 1:**
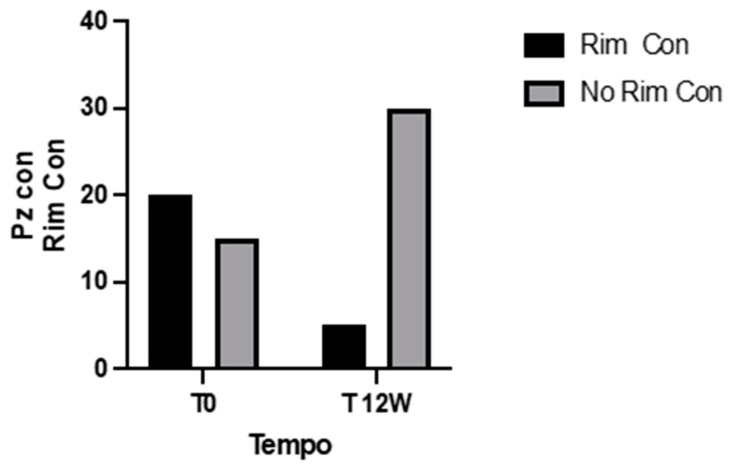
Contingency retrospective data (Fisher’s exact test).

Echocardiographic findings revealed that 57% of patients had concentric LV remodeling, and 14% had grade I diastolic dysfunction at T0 (Figure 1). These rates improved to 14% and 11.4%, respectively, at T1. Reductions in LVMI, RWT, and lateral E’ wave values indicated reverse remodeling and improved diastolic function (Table 4).

**Figure 2:**
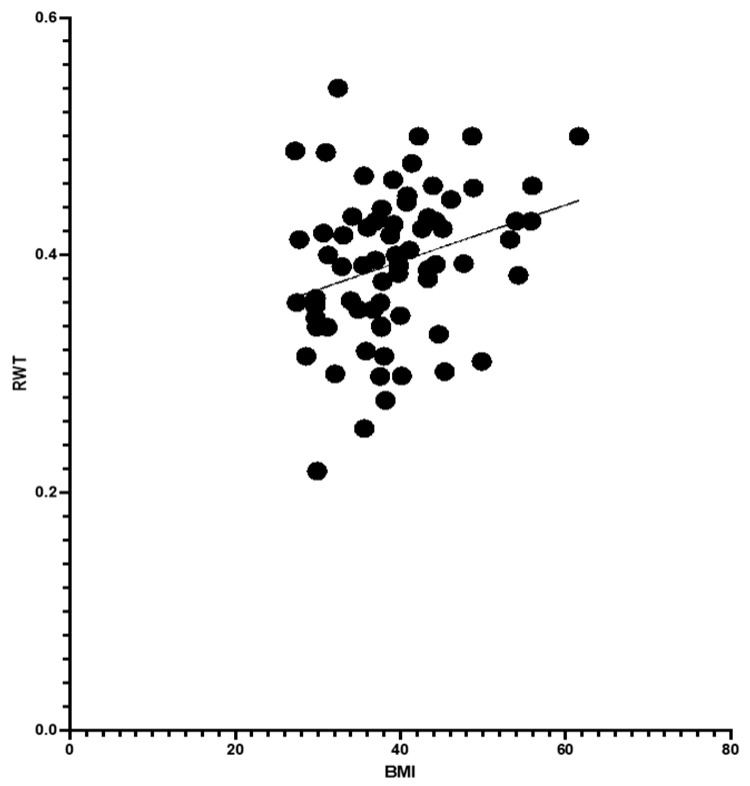
BMI and RWT with simple linear regression (*p*-value 0.0179 * and Pearson R 0.2822). “*” stands for statistically significant.

**Figure 3:**
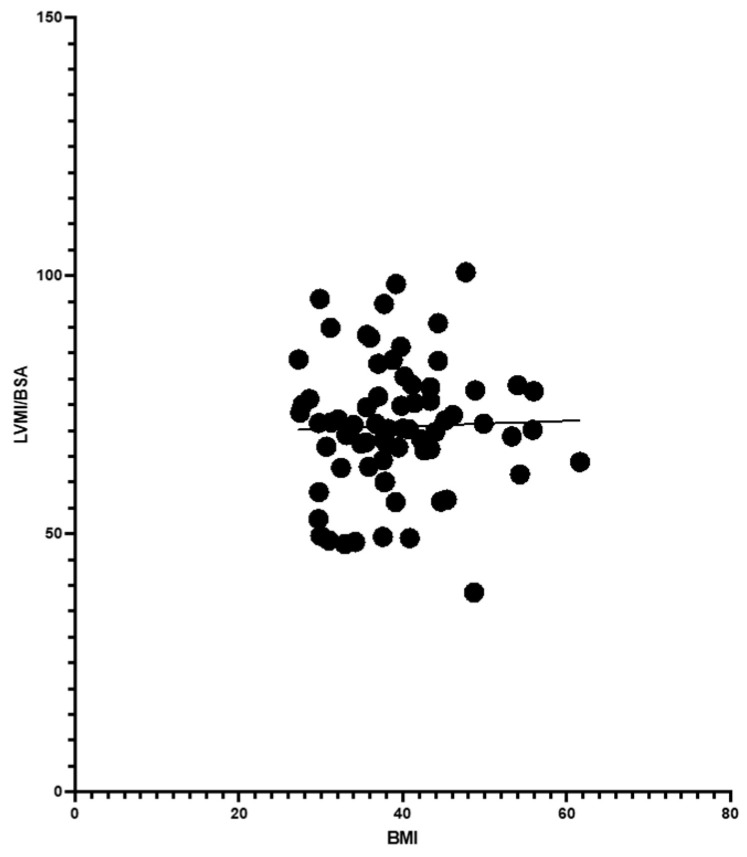
BMI and LVMI/BSA with simple linear regression (*p*-value 0.803 and Pearson R 0.030).

**Figure 4:**
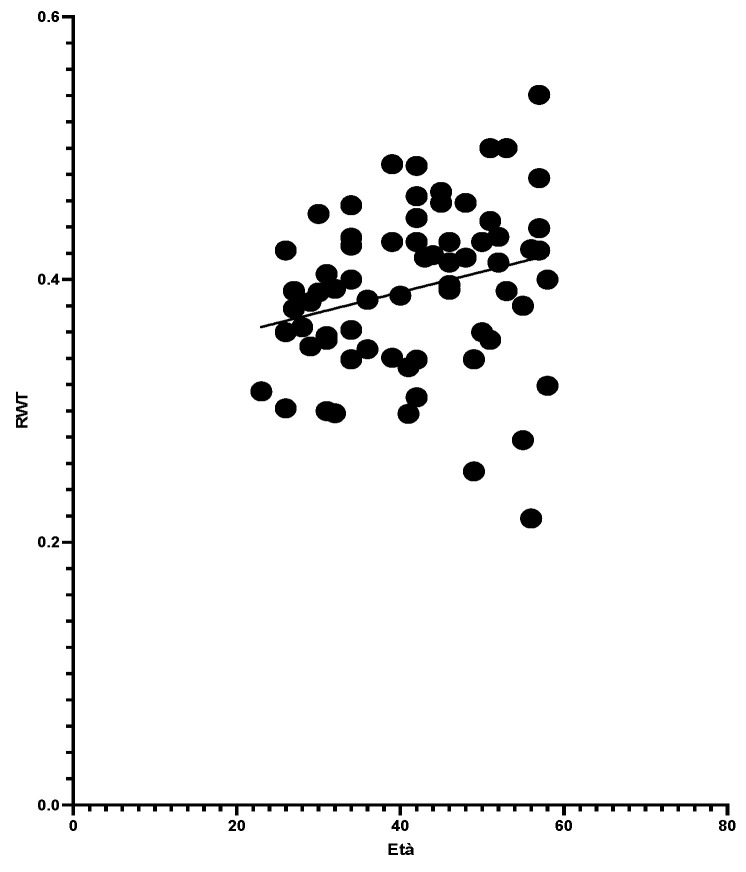
Age and RWT with simple linear regression (*p*-value 0.037 * and Pearson R 0.249). “*” stands for statistically significant.

Analysis showed BMI correlated positively with RWT, suggesting an impact of body weight on LV geometry. Age also moderately influenced RWT, though less significantly than BMI. However, BMI did not correlate with LVMI/BSA, indicating it was not a primary factor in LV hypertrophy (Figure 2, Figure 3 and Figure 4).

These results emphasize the rapid cardiovascular benefits of bariatric surgery, particularly in reversing concentric LV remodeling and improving metabolic health. However, the 12-week follow-up limits conclusions about long-term outcomes.

## 5. Discussion

Severe obesity is a major risk factor for cardiovascular diseases (CVDs), contributing to changes in cardiac structure and function even in individuals without a history of cardiovascular conditions. In this study, 71.4% of participants had undiagnosed cardiovascular conditions, while 82.8% had unidentified risk factors, such as elevated blood pressure (29%) and dyslipidemia (57%). Obesity-related lipid imbalances were evident, with 22.8% of patients exhibiting low HDL levels (<40 mg/dL) and 82.8% having elevated LDL levels (>100 mg/dL). These findings emphasize the importance of early cardiovascular screening in obese individuals [15,23,24].

Obesity-induced left ventricular (LV) remodeling often presents as concentric remodeling without hypertrophy, driven by increased preload, afterload, and myocardial dysfunction. At baseline, 57% of patients had concentric remodeling, and 14% showed grade I diastolic dysfunction. Weight loss following bariatric surgery, specifically sleeve gastrectomy, significantly improved cardiac structure and function. After 12 weeks, only 14% exhibited concentric remodeling, and diastolic dysfunction decreased to 11.4%. Significant reductions in interventricular septum thickness, posterior wall thickness, LV mass index (LVMI), and relative wall thickness (RWT) were observed, indicating reverse remodeling [25,26].

The mechanisms underlying these improvements include decreased mechanical load, improved metabolic flexibility, and reduced systemic inflammation. Weight loss alleviates myocardial stress, decreases plasma volume, and mitigates sympathetic activation. It also reduces levels of inflammatory cytokines like TNF-α and IL-6, oxidative stress, and myocardial fibrosis, enhancing myocardial compliance and diastolic function [27]. Improvements in insulin sensitivity further restore cardiomyocyte function, reducing lipid accumulation and improving calcium cycling for efficient myocardial contraction and relaxation [26,28,29].

Despite these benefits, the study’s short 12-week follow-up limits its ability to confirm whether these structural improvements lead to sustained cardiovascular benefits, such as reduced incidence of heart failure or CVD mortality. Longer-term studies are needed to evaluate these outcomes and understand the relationship between short-term reverse remodeling and long-term cardiovascular health [30].

This study’s findings emphasize the effectiveness of bariatric surgery not only for weight loss but also for cardiovascular health [31]. The observed reductions in blood pressure, heart rate, and lipid profiles highlight the procedure’s role in mitigating obesity-related CVD risk. However, the retrospective nature of the study and its small sample size limit broader applicability. Future research should involve larger patient cohorts with extended follow-up periods, potentially incorporating myocardial biopsies and advanced imaging to further elucidate the molecular mechanisms of reverse remodeling.

This study’s findings have important clinical implications, especially given the short-term nature of the 12-week follow-up. The observed improvements in left ventricular (LV) remodeling, including reductions in left ventricular mass index (LVMI) and relative wall thickness (RWT), suggest that even within a brief period, bariatric surgery can initiate meaningful reverse cardiac remodeling. This aligns with prior studies demonstrating that reductions in hemodynamic load can lead to structural and functional cardiac improvements. However, the short duration of follow-up limits the ability to fully evaluate whether these changes will translate into sustained long-term benefits, such as reduced incidence of heart failure or cardiovascular mortality.

Bariatric surgery provides substantial cardiovascular benefits in obese individuals, initiating meaningful reverse cardiac remodeling even in the short term. These findings highlight the systemic advantages of significant weight loss and emphasize the need for early cardiovascular assessment and long-term monitoring to optimize outcomes for patients undergoing bariatric surgery.

## 6. Limitations

While this study provides valuable insights into the effects of sleeve gastrectomy on obese patients, it is essential to acknowledge several limitations that may affect the generalizability and robustness of the findings.

The study’s retrospective and observational nature and the relatively small sample size of 35 patients limits the statistical power of the analysis. The study period of 12 weeks post-surgery, although useful for observing short-term effects, is insufficient to assess the long-term sustainability of weight loss and cardiovascular improvements. Although, the anthropometric changes in the study population are of great significance, the lack of a control group represents a further limitation. Furthermore, the study was conducted at a single center in Italy.

## 7. Conclusions and Future Perspective

Obesity is a chronic condition closely tied to cardiovascular risk factors and cardiac alterations. Bariatric surgery, particularly sleeve gastrectomy (SG), offers a powerful approach for mitigating these risks by achieving significant weight loss. This study demonstrates the potential of SG to improve anthropometric and cardiovascular parameters within a short 12-week follow-up, highlighting reductions in weight, BMI, and waist and hip circumferences, alongside better blood pressure, heart rate, and lipid profiles. Importantly, the reversal of concentric left ventricular remodeling and improvements in echocardiographic parameters, such as reduced left ventricular mass index (LVMI) and relative wall thickness (RWT), emphasize the cardiac benefits of weight loss.

Future research should focus on longer follow-up periods to confirm the durability of these benefits and their impact on cardiovascular outcomes, such as heart failure or myocardial infarction. Studies exploring additional cardiac parameters, including myocardial strain and diastolic function, are also needed. Investigating the molecular mechanisms behind these changes, such as neurohormonal modulation, inflammatory reduction, and metabolic improvements, could provide deeper insights.

In conclusion, SG facilitates substantial weight loss and meaningful cardiovascular improvements, establishing its critical role in managing obesity and its complications. Further research is essential to expand our understanding of the long-term benefits and mechanisms driving these outcomes.

## Figures and Tables

**Table 1:** Anthropometric and hemodynamic data at baseline (T0) and 12 weeks (T1).

	T0 (n = 35)	T1 (n = 35)	*p*-Value
Female	20 (57%)	=	ns
Age (years)	41.5 ± 10.3	41.8 ± 10.3	ns
Hip circumference (cm)	136.5 ± 19.13	118.2 ± 14.34	<0.0001 *
Waist circumference (cm)	127.7 ± 20.04	108.5 ± 14.51	<0.0001 *
Height (m)	1.68 ± 0.12	1.68 ± 0.12	ns
Weight (kg)	124.1 ± 23.47	99.6 ± 18.71	<0.0001 *
BMI (kg/m^2^)	43.5 ± 6.67	35.03 ± 5.91	<0.0001 *
BSA (m^2^)	2.29 ± 0.27	2.08 ± 0.24	<0.0001 *
Systolic blood pressure (mmHg)	126.5 ± 13	119.9 ± 11.41	0.0027 *
Diastolic blood pressure (mmHg)	82.29 ± 9.28	78.34 ± 5.98	0.0055 *
Heart rate (bpm)	78.26 ± 11.36	64.6 ± 8.51	<0.0001 *

“*” stands for statistically significant, “ns” stands for non significant.

**Table 2:** Medical history and risk factors.

	T0 (n = 35)	T1 (n = 35)
Family history of cardiovascular disease, n (%)	12 (34%)	-
Chronic coronary artery disease, n (%)	2 (5.7%)	-
Hypertension (mmHg)	8 (22%)	-
Elevated blood pressure values, n (%)	10 (29%)	1 (2.85%)
Total dyslipidemia, n (%)	24 (68.6%)	8 (22.8%)
Known dyslipidemia, n (%)	4 (11%)	-
Unknown dyslipidemia, n (%)	20 (57%)	4 (11%)
Type II diabetes mellitus, n (%)	0	0
High risk for diabetes mellitus, n (%)	12 (35.28%)	0
Hyperuricemia, n (%)	12 (34%)	0
Smokers, n (%)	7 (20%)	-
Ex-smokers, n (%)	3 (8.5%)	-

**Table 3:** Laboratory tests at T0 and T1.

	T0 (n = 35)	T1 (n = 35)	*p*-Value
Albumin (gr/dL)	4.29 ± 0.15	4.11 ± 0.46	ns
C-reactive protein (mg/L)	1.47 ± 1.11	5.41 ± 6.58	0.0105 *
Vitamin D (UI)	13.02 ± 5.06	14.18 ± 8.28	ns
Glycated hemoglobin (%)	5.92 ± 0.53	5.45 ± 0.44	0.002 *
Glucose (mg/dL)	98.77 ± 20.93	88.56 ± 12.67	0.0312 *
Blood urea nitrogen (mg/dL)	29.33 ± 6.41	29.21 ± 8.23	ns
Uric acid (mg/dL)	6.47 ± 1.27	5.89 ± 1.26	0.0307 *
Estimated glomerular filtration rate (mL/min)	131.3 ± 32.94	101.3 ± 20.44	ns
Creatinine (mg/mL)	0.77 ± 0.15	0.75 ± 0.18	ns
Sodium (mEq/L)	138 ± 1.35	141.6 ± 2.25	0.0002 *
Potassium (mEq/L)	4.22 ± 0.22	4.14 ± 0.31	ns
Bilirubin	0.67 ± 0.43	0.81 ± 0.33	ns
AST (aspartate aminotransferase) (U/L)	20.75 ± 8.18	19.25 ± 3.77	ns
ALT (alanine aminotransferase) (U/L)	24.33 ± 14.43	16.67 ± 4.51	ns
Calcium (mg/dL)	9.38 ± 0.29	9.77 ± 0.40	ns
BNP (B-type natriuretic peptide) (pg/mL)	19.78 ± 12.47	24.42 ± 8.9	ns
Total cholesterol (mg/dL)	191.9 ± 31.9	179.8 ± 24.64	ns
HDL-C (high-density lipoprotein cholesterol)	48.67 ± 9.55	53.67 ± 16.62	ns
LDL-C (low-density lipoprotein cholesterol)	124.2 ± 25.82	108.5 ± 21.69	0.0127 *
Triglycerides	95.8 ± 27.99	89.4 ± 25.36	ns
White blood cell count (WBC) (n/µL)	6.87 ± 1.75	6.45 ± 1.81	ns
Hemoglobin (g/dL)	13.46 ± 1.26	13.14 ± 0.89	ns
Platelets (n/µL)	254.9 ± 54.31	239.6 ± 54	ns

“*” stands for statistically significant, “ns” stands for non significant.

**Table 4:** Diastolic and systolic function in obese patients assessed by standard and advanced echocardiographic parameters.

	T0 (n = 35)	T1 (n = 35)	*p*
Ejection fraction (%)	61.99 ± 6.74	63.74 ± 5.81	ns
Ascending aorta (mm)	31.37 ± 3.18	30.86 ± 3.02	ns
PAPs (mmHg)	17.36 ± 5.52	18.68 ± 5.74	ns
IVSd (mm)	10.4 ± 1.19	9.48 ± 1.06	ns
LVPWd (mm)	8.97 ± 1.15	8.05 ± 1.55	0.0034 *
dVStd (mm)	47.63 ± 5.58	47.6 ± 5.55	ns
RA area (cm^2^)	13.7 ± 3.33	14.2 ± 3.34	ns
RWT	0.41 ± 0.05	0.37 ± 0.06	0.0023 *
E wave (m/s)	0.76 ± 15.45	0.75 ± 16.24	ns
A wave (m/s)	0.69 ± 18.12	0.66 ± 14.07	ns
E/A	1.16 ± 0.38	1.18 ± 0.31	ns
Deceleration time (ms)	213.5 ± 50.46	217 ± 51.67	ns
E/e’	6.74 ± 1.005	6.16 ± 1.54	0.0443 *
LAVi (ml/m^2^)	24 ± 6.13	24.61 ± 7.73	ns
TAPSE (mm)	24.43 ± 3.02	24.97 ± 3.16	ns
RVs’ (cm/s)	13.34 ± 1.86	13.17 ± 1.93	ns
LVMI/BSA	72.17 ± 13.07	69.45 ± 12.57	ns
LVESV (mL)	43.69 ± 15.89	40.91 ± 16.04	ns
LVEDV (mL)	113.7 ± 32.46	111.3 ± 36.33	ns
E’l wave (cm/s)	13.26 ± 3.56	15.14 ± 4.24	0.0002 *
E’s wave (cm/s)	9.8 ± 2.16	10.34 ± 2.66	ns
RVd1 (mm)	32.26 ± 3.49	30.86 ± 3.78	0.0080 *
LVMI (g/m^2^)	166 ± 38.98	145.7 ± 36.13	0.0002 *

LVEDV: left ventricular end-diastolic volume; LVESV: left ventricular end-systolic volume; IVSd: interventricular septum at end-diastole; LVPWd: left ventricular posterior wall at end-diastole; LV: left ventricle; RWT: relative wall thickness; LAVi: indexed left atrial volume; TAPSE: tricuspid annular plane systolic excursion; RV: right ventricle; LVMI: left ventricular mass index. “*” stands for statistically significant, “ns” stands for non significant.

## Data Availability

The data presented in this study are available on request from the corresponding author due to privacy.
